# Towards Inclusive Diagnostics for Neglected Tropical Diseases: User Experience of a New Digital Diagnostic Device in Low-Income Settings

**DOI:** 10.3390/tropicalmed8030176

**Published:** 2023-03-16

**Authors:** Adeola Onasanya, Michel Bengtson, Temitope Agbana, Opeyemi Oladunni, Jo van Engelen, Oladimeji Oladepo, Jan Carel Diehl

**Affiliations:** 1 Sustainable Design Engineering, Delft University of Technology, 2628 CE Delft, The Netherlands; 2 Centre of Infectious Diseases, Department of Parasitology, Leiden University Medical Centre, 2333 ZC Leiden, The Netherlands; 3 AiDx Medical BV, 2641 KM Pijnacker, The Netherlands; 4 Department of Public Health, Adeleke University, Ede 232104, Nigeria; 5 Department of Health Promotion and Education, College of Medicine, University of Ibadan, Ibadan 200132, Nigeria

**Keywords:** NTDs, diagnostics, end-user, user experience, Nigeria

## Abstract

Designing new and inclusive diagnostic tools to detect Neglected Tropical Diseases (NTDs) to achieve rational disease control requires a co-design process where end-users’ input is important. Failure to involve all potential end-users in new diagnostics for NTDs can result in low use and adoption failure, leading to persistent infection hot spots and ineffective disease control. There are different categories of potential end-users of new diagnostic tools for NTD control, and it is unclear if there are differences between the user efficiency, effectiveness, perception, and acceptability across these end-user categories. This study evaluated the usability, user perception, contextual factors affecting the user’s experience, and acceptability of a new digital optical diagnostic device for NTDs across three types of potential end users. A total of 21 participants were tested. Laboratory scientists, technicians, and Community Health Extension Workers (CHEWs) in training achieved similar scores on the usability and user perception questionnaires with no statistically significant difference between end-user categories. All participants also have high scores for the user perception domains which strongly correlate with the acceptability of the AiDx NTDx Assist device. This study indicates that, by providing digital diagnostic tools in combination with minimal training and support, CHEWs undergoing training and, by extension, CHEWs post-training, can be involved in the diagnoses of NTDs, potentially enhancing a community’s capabilities to diagnose, treat, and control NTDs.

## 1. Introduction

Neglected Tropical Diseases (NTDs) are a group of diseases found in tropical and subtropical regions of the world, especially in Africa, Asia, and Latin America [1]. NTDs are common in regions where access to clean water and adequate human waste disposal are limited with significant effects on the physical, social, and economic well-being of more than one billion individuals [2]. The limitations of current diagnostics for NTDs regarding performance and affordability have been highlighted in several studies [3,4,5], including ergonomic problems arising from the use of conventional microscopes [6].

To address these limitations, we designed a new diagnostic device to increase the screening of parasitic NTDs such as lymphatic filariasis, schistosomiasis, and onchocerciasis. This device, the AiDx NTDx Assist machine, is an automated digital microscope designed for the quick detection of parasites in blood and urine. It provides expert-independent analysis and could strengthen task-shifting programs such that non-experts, for instance, community healthcare workers, could be easily trained and empowered to perform quick sample screening for NTDs at the community and primary healthcare level. It has been validated for detection of lymphatic filariasis in Nigeria [7] in lab-based settings. Prepared slides are inserted into the device, patient data is input, and an automatic scan is started. The AiDx device has a processing speed of 10 min per sample, and the outputs of the scan are visually displayed and flagged when microfilaria is detected.

### 1.1. Designing for Neglected Tropical Diseases

Designing for the NTD space requires the involvement of stakeholders and end-users from the early stages of the design process in what is known as a human-centred approach to design [8]. Human factors play a critical role in the development and use of medical devices and diagnostics [9]. It is therefore important to co-create and improve devices by involving potential end-users in all stages of the design cycle, which include the specification, ideation, prototyping, and manufacturing process of diagnostic medical devices. One of the ways to improve the design, usability, and acceptability of a new NTD diagnostic device from a human-centred perspective is to evaluate the user experience by potential end-users.

User experience is defined as a user’s perceptions and/or responses resulting from the use or anticipated use of a product [10]. User experience research focuses on the dynamics of experience, personal characteristics, context, and product interaction. Components of user experience research can include usability (i.e., functionality and system performance), user perception (i.e., interactive behaviour, assistive capabilities arising from the users’ prior experiences, skills, attitudes, personalities, and abilities), and acceptability [11], all of which can be affected by contextual factors [12,13].

Usability is an important concept within user experience testing. It is the extent to which a product can be used by context-specific end-users to achieve specified goals [11]. Usability as an evaluation method is employed to redefine and improve a medical product during its development life-cycle, based on end-user requirements and needs. As such, usability testing can be conducted at one point during the product development cycle or at multiple points during, for example, prototype validation. Usability testing can also be conducted at the end of the product development cycle using both simulated and real-life settings [14]. The Systems Usability Study (SUS) is a validated scale that is commonly employed for testing product usability [15]; however, SUS has limited use for medical devices due to the absence of important factors, such as human technical performance. Human technical performance-based testing parameters include efficiency, effectiveness, error rates, and satisfaction [11,16], and it is different from device performance-based testing that focuses on sensitivity and specificity.

The selection of end-users is important for the validity of a usability study. Therefore, it is critical to identify and recruit representative users. The number of recommended end-users for usability testing ranges from 15 [17] to 25 [18]. However, the number of testers needs to be carefully selected since a meticulously crafted criterion for selection will bring greater returns on problem detection during usability testing.

Apart from performance-based usability testing, user perception and acceptability have been employed to evaluate user health interventions in healthcare [19]. User perception testing deals with gathering cognitive information based on interactive behaviour with the product. Several concepts, such as perceived ease of use, perceived usefulness, intention to use, and perceived ease of learning, have been used to assess user perception [14,20,21,22,23]. For diagnostic devices, particularly, perceived ease of learning is an important concept to explore, as a tedious multi-step use process can impede both the perceived usability and potential acceptability of the device.

In addition to user perception, the acceptability of a product will determine willingness to adopt and use the product. Successful implementation and scale-up of diagnostic device usage depend on the usability, user perception, and subsequent acceptability of the intervention to both healthcare managers, who are the decision-makers for medical device procurement, and end-users [24]. The Technology Acceptance Model (TAM) has been used as a guiding framework to determine acceptability. There is a strong correlation between user perception concepts and the acceptability of new devices [20,21,22,23,25]. In this context, the user perception of the medical device by end-users is a strong determinant of acceptability by end-users, and we will be inferring the acceptability of this device from the user’s perception of the device.

Although assessing acceptability is important for the subsequent use of diagnostic devices, it is also important to understand barriers to acceptability beyond the user’s perception of device usage. Other concepts, such as attitude to technology, trust, and contextual issues, can affect device acceptability [25] and should be elicited to give an all-encompassing assessment of acceptability.

**Figure 1:**
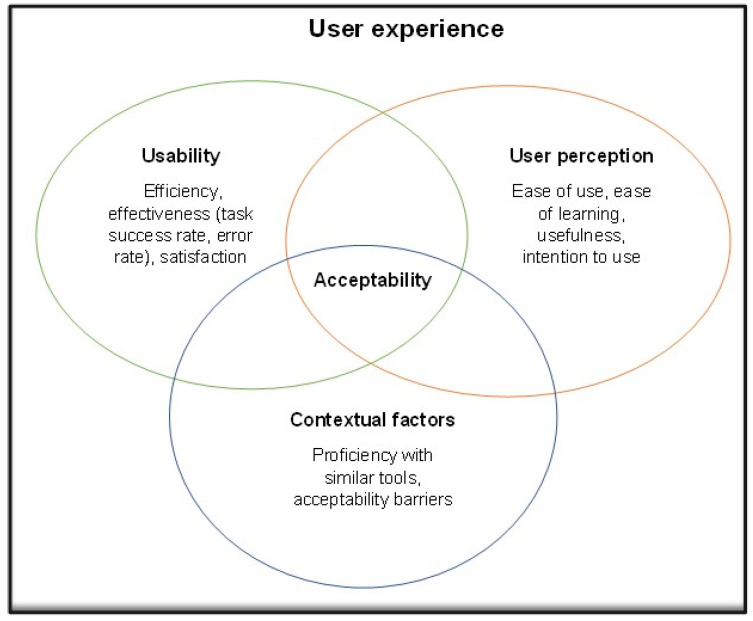
User experience conceptual diagram.

In this study, we researched/explored the following three themes: (1) usability in terms of efficiency, effectiveness, error rate, and satisfaction; (2) user perception in terms of perceived ease of use, perceived ease of learning, perceived usefulness, and intention to use; and (3) contextual factors in terms of barriers to acceptability and proficiency with similar devices, all of which collectively contribute to the overall user experience (Figure 1).

### 1.2. Problem Statement

Since NTDs are most common within Sub-Saharan Africa, it is imperative to carry out user experience testing of the AiDx NTDx Assist device within this context. It is known that contextual factors can affect the user experience and acceptability of medical devices [26], so the lack of contextual understanding of the use context of devices is likely to lead to underperformance and abandonment of medical devices. Medical devices are usually created in a high-income context, and technology transfer is not usually considered, leading to acceptability failures in Africa [27]. One way to ensure technology is transferred appropriately is through the application of user experience studies to gauge end-user experience with device usage and fit within the user context. Nigeria was selected for the user experience testing for two reasons: it has a large population, and NTDs are endemic within the country [28]. This makes the country an important and relevant testing context for the device.

### 1.3. Aim and Objectives of the Study

This study aims to assess the user experience of a working model of the AiDx NTDx Assist device for diagnosing NTDs by evaluating usability, user perception, and contextual factors that can affect acceptability based on end-user personae. The objectives of this study were: (1) to assess usability by observing participants during the use of the device and a post-observation questionnaire; (2) to assess user perception by the use of a post-observation questionnaire; and (3) to assess contextual factors that can affect acceptability by interviewing end-users through a semi-structured questionnaire. Several prior studies have evaluated the user context [5,29], use-case, end-user personae [30], end-user perspective [31], and performance [7] of novel digital diagnostic optical devices in Nigeria. This study also aims to test laboratory-based use-case scenarios for the AiDx Assist device before deployment for field-based use-case scenarios. Therefore, the results of this study will complement previous research efforts to ensure the fit, uptake, and use of new digital diagnostic tools in endemic settings.

## 2. Materials and Methods

### 2.1. Study Design

We used a moderated laboratory-based evaluative research approach focusing on the user experience aspects of the human-centred design methods.

### 2.2. Tools

**Figure 2:**
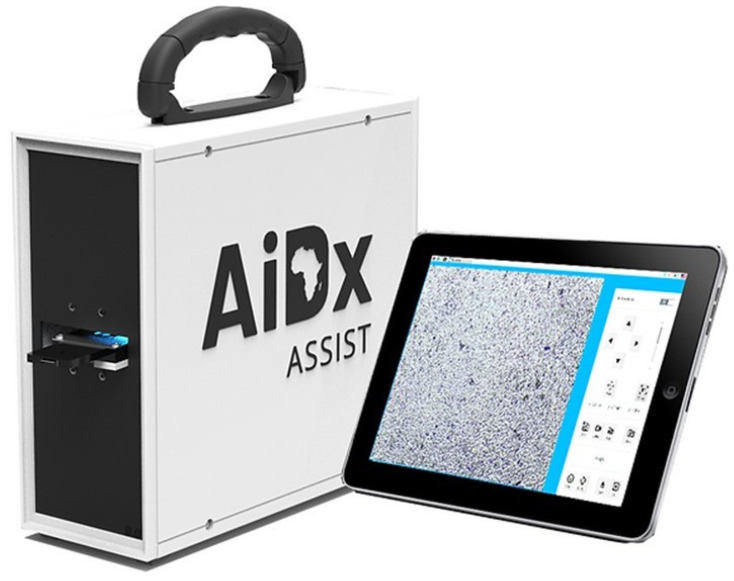
The AiDx NTDx Assist device.

Four tools were used in this study. These include (1) an instruction manual for use of the AiDx NTDx Assist device (Figure 2), (2) an observational checklist to be completed by a single investigator, (3) a semi-structured post-observational questionnaire, and (4) a user perception questionnaire to be completed by the participants.

The instruction manual depicts four steps taken in using the device: (a) turning on the machine, (b) starting the application, (c) inputting slides, and (d) reading output results. The observational checklist measures timing and documents consistency in the use of the four steps when using the device. The post-observational, semi-structured questionnaire focuses on the overall experience with the task given, satisfaction, trust, and aesthetics.

The user perception questionnaire is a semi-structured questionnaire with three sections. Section 1 elicits proficiency with the devices. Section 2 focuses on user perception and contains 42 items within four distinct perception domains: (i) perceived ease of learning, (ii) perceived ease of use, (iii) perceived usefulness, and (iv) intention to use, on a five-point Likert scale (from 1—Strongly disagree to 5—Strongly agree). Section 2 is based on the work done by Parreira et al. (2020) which tests users’ utilization of new technologies by focusing on interactions between functional and behavioural aspects. Parreira’s scale was modified by reducing the questionnaire from a seven-point Likert scale to a five-point Likert scale to make the decision-making less confusing, increase the response rate, and provide a comparable scale to the SUS without data extrapolation. We also modified statements within selected domains to reflect our device’s focus on NTDs and the healthcare context in Nigeria. Section 3 elucidates potential barriers to acceptability and suggestions to improve device acceptability. Data was captured using the Qualtrics software.

### 2.3. Study Participants and Ethics

Study participants’ selection was based on data from user personae research [29,30] and included laboratory scientists, technicians, and Community Health Extension Workers (CHEWs) who are currently undergoing training (Table 1). We used second-year CHEWs in training as a proxy for CHEWs because invited CHEWs could not leave primary care centres to visit the testing laboratory due to health worker shortages. We infer that CHEWs in training perform worse or similarly to CHEWs in practice. All participants have prior knowledge and or experience with NTD diagnosis.

The study was approved by the UCH/UI Joint Ethical Review Committee, College of Medicine, University of Ibadan. (Reference: UI/EC/21/0641). All participants agreed to the data collection and signed informed consent forms.

### 2.4. Procedure

Participants were taken through a short demonstration and training (of about one hour) on the use of the AiDx NTDx Assist device using the instruction manual (Tool One). After training, participants were asked to use the device based on what they learned from memory (i.e., without the aid of the user manual) under the observation of a single investigator (Tool two: observational checklist). The observational checklist captures four steps taken in using the device: (a) turning on the machine, (b) starting the application, (c) putting in slides, and (d) reading output results. The device scanning time was programmed for 8 min and not captured by the checklist since it is constant across all participants. Thereafter, participants were asked to fill out the questionnaires (Tools three and four) to document their experience.

### 2.5. Data Analysis

Qualitative data were analysed by content analysis to identify end-users’ opinions on acceptability barriers, trust, satisfaction, and aesthetics. For the quantitative data, descriptive statistics were generated and non-parametric tests such as the statistical test of percentages and Mann-Whitney U tests were carried out using Python software.

## 3. Results

### 3.1. Usability Results

Using the observation checklist (Tool two); the following results were obtained.

#### 3.1.1. Efficiency

Efficiency is the total time a user needs to complete the task successfully. Four tasks were assigned in this study: turning on the device, starting the application on the device, putting a slide into the device, and reading the device output. All participants had an average of 2.4 s, 2.8 s, 4.3 s, and 7.2 s across all tasks with variation across the different groups of participants (See Table 2). On average, the CHEWs in training spent less time per task than other groups.

#### 3.1.2. Effectiveness (Task Success Rate and Error Rate)

Effectiveness gives insight into the number of completed successful tasks without support from another human or the manual (task success rate), as well as the error rate. In this study, the task success rate was 100%. The error rate was calculated based on the number of deviations from manual instructions. For instance, pressing the wrong button for opening the device was considered an error. The average error rate was 33% across all participant groups with inter-group variations. Laboratory technicians had the largest error rate (87.5%) compared to CHEWs in training (2.3%) (See Table 2). The most common error noted was starting the application, followed by putting in a slide.

#### 3.1.3. Satisfaction

Satisfaction data was collected using the semi-structured post-observational questionnaire (Tool three). Satisfaction is a broad category which includes domains such as overall experience, satisfaction with features, user trust, and others. In this study, all participants rated their experience as positive, and most were satisfied with the features, especially the visual output which made diagnosis easy. Most participants (20/21) rated the device as reliable for the diagnosis of NTDs. All participants (100%) commented positively about the device’s appearance; however, three of 21 participants (14.3%) wanted the device to be smaller than the current size. Eighteen participants (85.7%) stated that the device could be used to support quality control checks of microscopy results.

### 3.2. Contextual Factors

Using Tools three and four, this part of the survey was used to assess contextual factors, such as proficiency with similar digital tools and acceptability barriers.

#### 3.2.1. Proficiency with Similar Digital and Optical Devices

**Figure 3:**
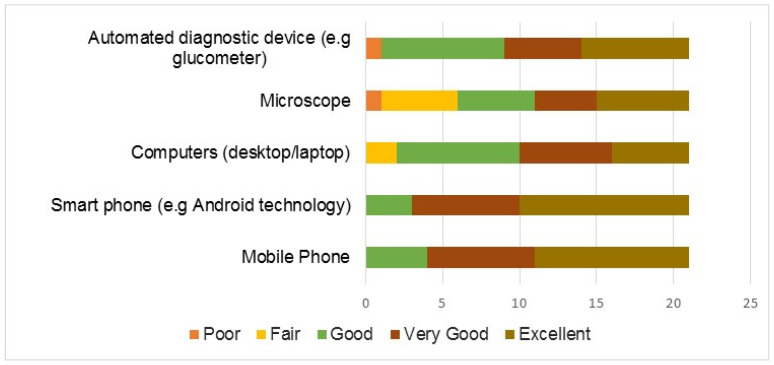
Proficiency rating with digital/optical devices by respondents.

At baseline, all participants had used or had been previously taught how to use the listed devices in Figure 3, so we assessed the extent of proficiency with the devices. Results showed that most of the participants rated their proficiency with similar digital and optical devices as good. Less than 5% of participants had low levels of proficiency with the glucometer and microscope (Figure 3).

Further analysis was done to assess the association between proficiency with the listed digital and optical tools and sociodemographic data. There was an association between the proficiency rating of optical/digital devices with years of experience as seen in Table 3. Participants whose level of training was higher or equal to laboratory technologists/technicians had a higher user proficiency with tools such as smartphones, optical devices, and automated diagnostic devices. Participants with greater than or equal to 10 years of work experience had a higher user proficiency with optical devices such as microscopes.

#### 3.2.2. Acceptability Barriers

We also studied potential barriers to acceptability and ways to improve device acceptability. Regarding potential barriers to acceptability, ten respondents (47.6%) mentioned problems with electricity supply hampering the use of the device and suggested the incorporation of alternate sources of power, such as solar energy, if the device will be used in primary healthcare settings. Four respondents (19%) mentioned challenges with sourcing a separate computer monitor to view the results of the device output. They suggested that the device be interoperable by a mobile phone and/or android computing system which is readily available. One person (4.8%) mentioned the importance of acceptability by gatekeepers, such as the medical laboratory science associations, leading to better acceptability by laboratory scientists.

### 3.3. User Perception

Using Tool four, the user perception questionnaire tested four domains: perceived ease of learning, perceived ease of use, perceived usefulness, and intention to use. The mode of all the variables was five. The median was five for most of the variables, except for the ease of use which gave a value of four, indicating a good user experience scoring. The mean score was above four across the user perception domains. Cronbach’s alpha was calculated to measure the questionnaire’s internal consistency. The Cronbach’s alpha of all items was 0.9248, showing a high level of reliability of the questionnaire as seen in Table 4.

Most of the respondents had a high level of scoring for each variable category. Ease of use and ease of learning had the highest variability. However, the variability noted is from a few outliers in the consensus scores for these two domains. The variability does not have a significant effect on the study outcomes. The usefulness and intention to use categories have the least variability in scoring.

We further carried out inter-group analysis using the Mann-Whitney U test to identify the differences between the dependent variables. These are the user perception domains and the independent variables which are the sociodemographic characteristics (Table 5). Results show no difference between the categorical groups highlighted in Table 3 and the user perception domains.

## 4. Discussion

This study aimed to evaluate the user experience of a new diagnostic device for NTDs within the sub-Saharan African context using identified potential end-users. To the best of our knowledge, a literature search for publications on the usability of medical devices within the Nigerian context did not yield any significant results. However, we found studies on digital health diagnostics for detecting NTDs [32,33,34] but only one study protocol [35] on user experience of a digital health diagnostic for skin NTDs within the African context.

Developing a medical diagnostic device requires the input of end-users. Involving end-users enables early identification of user needs and contextual requirements. A user experience study is important because it ensures that the prototypes and the final product meet end-user requirements. The user experience of a device can also determine its acceptability by end-users, which determines uptake and continual use. Many factors, such as user efficiency, effectiveness, and satisfaction, are important to consider for the usability aspects of the user experience study. In this study, the average user in CHEW training (4.2 s) was faster than laboratory scientists (4.5 s), with a similar pattern seen with the error rates, indicating that a faster time did not correlate with increased errors. The slightly faster speed of task completion by users in CHEW training may be due to faster reflexes due to the younger age group of the CHEWs in training. Laboratory technologists/technicians were slower and had a substantially higher error rate compared with laboratory scientists. Some studies point to limited experience with digital tools as a probable cause [36,37], although laboratory technicians indicated a high level of experience with digital tools in our study (Table 3). There were few laboratory technicians in the study sample (Table 1) which has likely amplified the error rates from one individual in the group. It is therefore likely that the efficiency rates and error rates may improve if more laboratory technologists/technicians were included in this study. However, despite these factors, all categories of participants were able to complete the tasks within 4.2 s, which generally indicates that the design of the device allowed ease of use and consequent efficiency, thereby contributing to a positive user experience. In addition, most participants were satisfied with the device, contributing to a positive user experience and positively impacting the intention to use the device.

To use diagnostic devices such as the AiDx NTDx Assist device, it is important to consider other contextual factors, such as the proficiency of respondents with the use of devices that have similar characteristics. Considering contextual factors can be used to support the data and validate the results of the usability testing. In this study, the results of testing for proficiency with some medical and optical devices are similar to the output of the user perception questionnaire (Figure 2). This finding suggests that end-users who have interacted with medical devices or medical technology are likely to find other medical devices with similar characteristics that are easy to use. This familiarity may have a positive effect on the acceptability of similar new devices. In addition, the difference between experiential ratings for proficiency with similar digital tools (Table 3) did not have a direct relationship with the usability scores. This result indicates that prior exposure (training or experience-based) with similar tools with additional training is sufficient for high proficiency in the use of digital devices by the lowest end-user.

Other contextual factors explored in this study include barriers to acceptability. Technical infrastructure, such as availability of computer hardware, device interoperability with android technology, and electricity was highlighted as contributing to the barriers to the acceptability and use of the devices. This finding signifies that the actual use of the device use is also dependent on other non-human factors which may be a strong determinant of device abandonment, despite the positive user experience recorded.

We used a mix of end-users in this study to simulate the pattern of users of the device at the primary healthcare level. There were no significant differences between the user perception scores of the different end-users, signifying that the device is easy to use and can be used by the lowest cadre of healthcare workers. In addition, the data presented in this study indicate that the testing of similar devices may not necessarily require highly-skilled workers such as laboratory scientists. CHEWs-in-training and, by extension, CHEWs are a reliable testing group with scores similar to those of laboratory scientists.

The following user perception domains were assessed: perceived ease of learning, perceived ease of use, perceived usefulness, and intention to use. These factors cover important aspects related to the acceptability of the device and can also be a strong determinant of the acceptance and use of a medical device [9,14,27]. The perceived ease of use, usefulness, and intention to use are strong determinants of acceptability [20,21,22,23,25], and the high scores for these domains suggest a high level of medical device acceptability by the end-users.

Devices that require minimal training are easy to learn and are a positive predictor of ease of use and acceptability, while the usefulness domain also strongly predicts acceptability [9,20]. The usefulness domain signifies the importance of the new device in the daily activities of the end-user. All the end-users agreed that the device was useful to their work, and they were willing to use and recommend the medical device for use to their colleagues.

There are some limitations to the study. First, the inequality across tested groups may account for the variation in usability scores. For instance, a larger group of laboratory technicians may elicit a lower error rate and higher efficiency. Second, the study obtained results based on first-time use, and repeated use and testing will likely increase efficiency and effectiveness and reduce error rates across all groups.

## 5. Conclusions

Our findings have practical implications for NTD management. First, diagnostic devices must be accessible at the primary care level for early detection and treatment. Our study provides evidence that CHEWs can readily and effectively use digital diagnostic devices in the NTD context. Second, innovative medical devices suitable for the context of use are necessary to meet WHO targets for NTD control and elimination. Last, device suitability can be assessed through user experience studies in addition to performance metrics. Involving potential end-users in NTD diagnostic tool development reduces the risk of desertion, increases awareness, and aids early detection and treatment, particularly in low-resource settings.

## Figures and Tables

**Table 1:** Sociodemographic characteristics of the participants (n = 21).

**Sex**	**Count**
Female	13
Male	8
**Education**	
Postgraduate	5
University Graduate (B.Sc.)	3
Diploma (OND/HND)	1
Health Technology School	12
**Years of Experience**	
0–4	11
5–9	2
10–14	3
15–19	2
20–24	2
≥25	1
**Level of Training**	
Community Health Extension Worker (CHEW) in-training	11
Laboratory Technologist/Technician	3
Laboratory Scientist	7

**Table 2:** Descriptive statistics for assigned tasks.

Category	Turning on the Device (s)	Starting the Application (s)	Putting in a Slide (s)	Reading Result Output (s)	Average Time (s)	Average Error Rate (%)
CHEW in training	2.5 ± 0.7	3.8 ± 1.4	2.5 ± 1.0	6.5 ± 4.9	3.3 ± 2.0	2.3%
Laboratory Technician	2.0 ± 0.0	4.5 ± 0.7	4.0 ± 0.0	8.5 ± 2.1	7.7 ± 0.7	87.5%
Laboratory Scientist	2.5 ± 1.0	4.8 ± 3.2	2.8 ± 1.4	8.0 ± 3.9	4.5 ± 4.6	9.3%
All participants	2.4 ± 0.8	2.8 ± 1.2	4.3 ± 2.2	7.2 ± 4.3	4.2 ± 2.1	33%

**Table 3:** Association between sociodemographic characteristics and proficiency with digital and optical tools.

Proficiency in Digital/ Optical Tools	Gender	Level of Education	Level of Training	Years of Experience
Mobile phone	0.83	0.69	0.07	0.58
Smartphone (Android technology)	0.95	0.64	0.01	0.52
Computer (desktop/laptop)	0.27	0.14	0.51	0.60
Optical devices (microscope)	0.11	0.14	0.00	0.01
Automated diagnostic device (e.g., glucometer)	0.52	0.08	0.01	0.22

**Table 4:** User perception domain summary statistics and reliability testing.

Perception Domain	Mean ± S.D	Median	Mode	Cronbach’s Alpha (All Items)
Ease of learning	4.1 ± 1.2	5.0	5	0.9248
Ease of use	4.3 ± 0.8	4.0	5	
Usefulness	4.6 ± 0.5	5.0	5	
Intention to use	4.6 ± 0.5	5.0	5	

**Table 5:** Mann Whitney U test comparing inter-group characteristics.

Variables/Domains	Ease of Learning	EASE OF USE	Usefulness	Intention to Use
Gender	W = 54.5, *p* = 0.85	W = 50.5, *p* = 0.90	W = 63.0, *p* = 0.34	W = 47.0, *p* = 0.66
Highest level of education	W = 32.5, *p* = 0.10	W = 39.0, *p* = 0.23	W = 49.5, *p* = 0.70	W = 63.0, *p* = 0.44
Level of training	W = 48.5, *p* = 0.67	W = 43.5, *p* = 0.41	W = 74.0, *p* = 0.085	W = 74.0, *p* = 0.08
Years of Experience	W = 40.0, *p* = 0.35	W = 53.5, *p* = 0.93	W = 51.5, *p* = 0.97	W = 46.0, *p* = 0.60

W = test statistic; *p* = *p*-value.

## Data Availability

The datasets generated and analysed during the current study are not publicly available but are available from the corresponding author upon reasonable request.
